# State Control of Medical Practice

**Published:** 1897-04-10

**Authors:** 


### STATE CONTROL OF MEDICAL PRACTICE.

Someone has said that the price of liberty is eternal vigilance, and there can be no doubt that unless the medical profession and those who represent it keep constantly on the watch, and are prepared to resist encroachment whenever any attempt is made to infringe on its liberty or restrain its freedom, we may soon find ourselves hampered in many unexpected directions in our efforts to do what is our first duty, viz., to cure our patients.

As an illustration of the sort of things that may be put upon us we may recall the obligation to report cases of plumbism and other diseases to the inspectors of factories, which is placed upon medical men by the Factory and Workshops Act, 1895. This, however, is but a trifle in comparison with what may be done to worry medical men, and bring them under subjection to municipal and other authorities, by means of clauses inserted into Bills which, on the surface, do not appear to have any direct connection with medical affairs.

As an instance of this we would draw attention to a discussion which took place at the New York Academy of Medicine on March 18th, on the occasion of the presentation of the report of a committee which had been appointed to consider the clauses having reference to the health department, which formed part of the proposed charter of the Greater New York.

Dr. Piffard and Dr. Jacobi drew attention to the following two extraordinary clauses in the proposed charter:—

"Sec. 1,248. The department of health may require any physician to subscribe, not less than three hours after service of a demand thereof upon him, an affidavit stating therein whether he has or has not any patient, who, in his opinion, shall then be sick of any pestilential, contagious, or infectious disease, and if he has any such patient to state in such affidavit his or her name and the house or place in the said city where he or she shall then be, and the nature or name of such disease, to the best of his knowledge and belief.

"Penalty for failing to report.

"Sec. 1,249. Every practising physician who shall refuse or neglect to perform the duties enjoined on him by the foregoing section shall be considered guilty of a misdemeanour, and shall also forfeit for each offence the sum of 250 dols. to be sued for and recovered by the health department. It shall be the duty of each visiting hospital and consulting physician to make an immediate report to the department of health of the name of every practising physician by whom we shall have reason to believe the provisions of the said section have been violated; and if such physician shall neglect or refuse to perform his duty the department shall order him to be suspended from any office he may hold, and he shall, moreover, be liable to such further penalty and to such prosecution for his violation of this law and of his duty as the Board of Health shall determine."

According to the New York *Medical Record*, from whose report we quote the above, Dr. Jacobi maintained that this turned physicians into spies. "It was undignified, unworthy and dangerous, and certainly ought to be struck out. It was moreover unconstitutional. By law physicians were certainly compelled to report a felony, but by this section they were required to report a misdemeanour, which was not in accord with the constitution."

In regard to this it is worth noting that we in England have not even a constitution to appeal to. If a law like this were once passed, law it would remain until repealed. Now comes the curious thing. It appears from what was said by the president, Dr. Janeway, that this has actually been the law for about eight years, and has been simply adopted from the present regulations of the board of health into the charter of the Greater New York. He said he did not know just how it had come to be inserted, but he thought it was from failure on the part of some physician, some eight years ago, to report a case of typhus fever or cholera. The law might look more aggressive than it was, for he knew of no instance in which it had been abused or enforced.

To this Dr. Jacobi rightly retorted that if the law was a dead letter it had better be struck out, for if it should be inserted into the new charter it might become very dangerous.

It appeared from the remarks of other speakers that some of the extraordinary powers which it was proposed to give to the health board had really been already given them by law, and had been in operation for 30 years. They had been tolerated only because the health department had been chary in their use, reserving their exercise for times of emergency. The committee did not recommend any change in the proposed charter, believing that the conservative influences which had prevailed in the past would continue, and that if at any time there should be any unwarranted exercise of such powers a re-action would take place which would bring about legislation which would again limit the powers of the health board.

We cannot, however, but see how dangerous is the power placed in the hands of a public body largely dependent for its popularity upon the fuss that it can make in times of panic and epidemic, and we think that the medical profession on this side of the water will be well advised to resist all encroachments which tend to the imposition of any such a tyranny over them as is implied by the existence of such regulations, unused though they be. Nothing can be more offensive and liable to abuse than that a public body should be allowed, at its own discretion, to bring into force laws which would not be tolerated if constantly in operation. Such a power is sure to become a mere engine of tyranny.

Dr. Quimby drew attention to the absolute power now given to the board of health. "They were given power to take charge of all hospitals for pestilential, contagious, and infectious diseases, and physicians might look forward to the time when they would gather up patients with venereal and most other diseases and conduct them to hospitals under their exclusive charge." We repeat our text, and say that the price of liberty is eternal vigilance.

